# Spatiotemporal optical vortex reconnections of multi-vortices

**DOI:** 10.1038/s41598-024-54216-4

**Published:** 2024-03-06

**Authors:** Jordan Adams, Imad Agha, Andy Chong

**Affiliations:** 1 Deparment of Electro-Optics and Photonics, University of Dayton, Dayton, OH 45434 USA; 2 Optics and Photonics, Riverside Research Institute, Beavercreek, OH 45431 USA; 3 Deparment of Physics, University of Dayton, Dayton, OH 45434 USA; 4 Department of Physics, Pusan National University, Busan, 46241 Republic of Korea; 5 Institute for Future Earth, Pusan National University, Busan, 46241 Republic of Korea

**Keywords:** Optical physics, Ultrafast photonics

## Abstract

Vortex reconnections are ubiquitous events found in diverse media. Here we show that vortex reconnections also occur between spatiotemporal vortices in optical waves. Since vortices exhibit orbital angular momentum (OAM), the reconnections of optical vortices create a variety of connected OAM states. Dispersion and diffraction can cause different reconnection pairs, depending on the orientation of the vortices. The transverse crossing of two vortices with a topological charge of one can produce unique vortex loop reconnection patterns. Higher topological charges result in arrays of vortex loops and connection points. Crossing of three vortices produces spherical structures made of three symmetrical vortex arms. A three vortices reconnection with higher topological charges develops complicated patterns similar to turbulence cascade phenomena in other media. Studying optical vortex interactions may bring insight into vortex reconnections in other fields. We also provide experimental results of two-vortex loop interaction.

## Introduction

Vortex reconnections are events found in viscous fluids^1–5^, superfluids^6,7^, Bose–Einstein condensates^8^, liquid crystals^9^, superconductors^10,11^, and magnetohydrodynamics^12^. Two vortices approach each other and at the time and place of the intersection, they simultaneously split and reconnect with the other vortex. Reconnections can drive turbulent phenomena in superfluids^6,7^ and viscous fluids such as aeroacoustic noise^1^. In plasmas, magnetic reconnections accelerate charged particles and are behind solar flares as well as magnetospheric substorms^12^.

Vortex reconnections in waves and optics have also been a topic of interest. Complicated three-dimensional vortex lines can occur in light^13–16^. Experimentally, this can occur by using spatial light modulators (SLM) to apply a phase mask to an optical beam and diverging or focusing the light. The designed vortex trajectories appear at a certain propagation distance. Even though complicated patterns can be generated, the vortices are stationary and there are no reconnection events. In one theoretical study, Berry and Dennis^16^ showed a variety of reconnections of vortex lines of complex scalar wavefunctions such as optical vortex lines. Among them, in a peculiar type of reconnection event, two reconnection points appear simultaneously while the two points are connected by a loop structure in optical waves^16^.

In this work, we show spatiotemporal optical vortex (STOV) reconnections. STOVs are a new type of optical vortex with transverse OAM which can be experimentally made by with several setups such as a pulse shaper to modulate the spatiotemporal phase of a pulse^17^, as well as nonlinear^18^ or time-reversal mechanisms^19^. In our work, we generated STOVs by the pulse shaper method initially, but other vortex states are created by propagation.

When STOVs are combined with simple spatial vortices in an optical wave packet as perpendicular lines, vortex reconnections appear with propagation. This is quite similar to reference 16 but differs in many aspects. While reference 16 discusses how the spatial vortices in monochromatic waves evolve, our work is based on polychromatic spatiotemporal waves with perpendicular vortices made of STOVs and spatial vortices. By combinations of focusing and propagation through dispersive media, reconnections can naturally evolve over time like reconnections in fluids. The reconnection process transforms the two line vortices into a unique loop structure with intersection points that connect one vortex to two vortices, creating a superposition in the vortex path. Since such vortices carry OAM, reconnections of vortices show interesting connections of OAM as well. In contrast to the spatially confined loop reconnection^16^ mentioned above, we also show higher topological states reconnect into arrays of loops.

With linear propagation of optical waves, we can even easily investigate three-vortex reconnections. Three-vortex reconnections have yet to be demonstrated in the field of optics or even other areas of physics. Despite performing only linear propagation, cascading nested vortex loop structures begin to emerge at a large topological charge. This shares a similarity to turbulence cascades between reconnected vortices in real fluids^2^. Experimental results are shown for the two-vortex loop connection. We strongly believe that studying these vortex reconnections in linear and scalar spatiotemporal optics will offer insight into vortex reconnection in other areas.

## Results

### Two-vortex reconnections

Generating spatiotemporal optical vortices typically involves applying a phase in the spatial-frequency and frequency domain (*k*_*x* _− *ω*) of a wavepacket. Exiting a pulse shaper and propagating a sufficiently far distance performs a 2D Fourier transform so that the desired spacetime structure (x − t) can be designed by calculating the necessary Fourier domain phase. Limited types of three-dimensional (3D) wave packets can be obtained by performing sequential 2D spatial-frequency and frequency phase modulations in orthogonal planes (i.e. *k*_*x*_ − *ω*, *k*_*Y*_ − *ω*, *k*_*x*_* –  k*_*Y* _) where the final results can be calculated with 3D Fourier transform. While diffraction causes the spatial-frequency spectrum at one plane to be mapped onto the spatial domain at a different location, insufficient propagation leads to an in-between state described by the Fresnel transform. Additionally, for pulses, acquiring a quadratic frequency phase from dispersive media can also be modeled with a Fresnel transform. To accurately model spatiotemporal optical vortices under variable propagation conditions, 3D (*k*_*x*_ − *k*_*Y*_ − *ω*) Fresnel transforms are needed. Using these transforms, we will eventually show that propagating through dispersive media or through a focus can cause vortex reconnections.

We start from two perpendicularly intersecting vortices embedded in a wave packet in the frequency domain and then examine the evolution in the space–time domain. For the wave packet, we start with an amplitude envelope that is Gaussian in both space and time (*x*, *y*, *t*) which corresponds to Gaussian in spatial-frequency and frequency (*k*_*x*_, *k*_*y*_, ω). We can add two vortices, one is a STOV while the other one is a regular spatial vortex, in the frequency domain and perform the Fresnel transform as shown in Eq. (1).

1 $$E = {\mathcal{F}}_{3D} \left\{ {\left( {ik_{x} + d\sqrt 2^{{l_{1} }} \omega } \right)^{{l_{1} }} \left( {k_{x} + \sqrt 2^{{l_{2} }} ik_{y} } \right)^{{l_{2} }} \exp \left( { - \left( {\frac{{k_{x} }}{w}} \right)^{2} - \left( {\frac{{k_{y} }}{w}} \right)^{2} - \left( {\frac{\omega }{\Delta \omega }} \right)^{2} - i\pi A\left( {k_{x}^{2} + k_{y}^{2} + \omega^{2} } \right)} \right)} \right\}$$

where ℱ_3*D*_ represents the 3D Fourier transform. The coefficient *A* is used to control the transformation from the frequency to the space–time domain, *d* = *w*/Δω is a factor to account for the frequency/spatial frequency bandwidth mismatch, and the topological charges (TC) of *l*_1_ and *l*_2_ are both set to one for now. If *A* is zero, then this is a standard Fourier transform while large positive *A* represents an inverse Fourier transform, bringing the function back to the *k*_*x*_ − *k*_*y*_ − ω space. Decreasing from a large positive value represents the focusing process of the wave packet in both space and time. At *A* = 0, the wave packet reaches the focus and diverges again as *A* becomes a negative number. Therefore, by tracking the wave packet with monotonically decreasing *A*, we can observe the vortex behavior around the focus. The advantage of this model is to observe the vortex reconnection behavior with only one parameter (*A*) which governs converging/diverging of the wave packet in space and time simultaneously. In fact, the spatial and temporal profiles can be controlled separately which will be discussed later.

**Figure 1:**
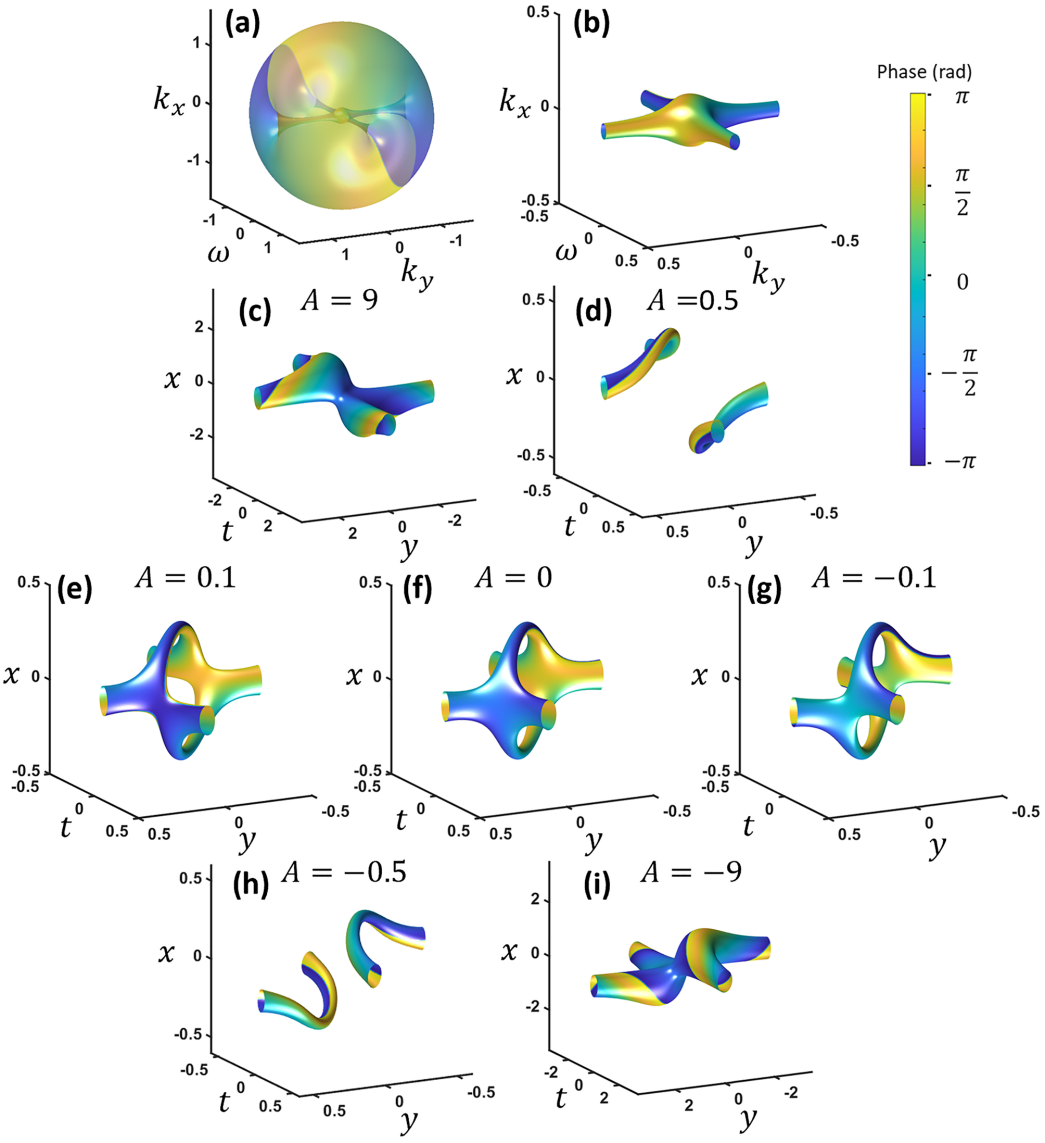
(**a**) The two vortices intersecting in the frequency domain shown outside and (**b**) inside the wavepacket envelope (**c–f**) The space–time domain starting from large *A*, and decreasing from left to right, top to bottom. Phase is shown from − π to π. ${k}_{x}$ and ${k}_{y}$ units are in inverse millimeters and $\omega$ units are in THz, while *x* and *y* units are in millimeters and *t* is in picoseconds.

Figure 1a shows iso-intensity profiles of the frequency domain with the two orthogonal vortices embedded in a wave packet. The phase is plotted on the iso-surface with phase from − π to π. Figure 1b is the view from inside the wave packet which gives a better perspective of two crossing vortices. Figure 1c–e shows the propagation in the space–time domain by performing the Fresnel transform. Each vortex causes a π-phase shift across the center of the other vortex. This makes the crossing vortices to break up while forming a phase matched continuous connection at 90° to the other vortex (Fig. 1c). With propagation (decreasing *A*) the vortices move further away from each other (Fig. 1c,d).

In the next phase, the vortices are drawn back toward each other while the middle sections reconnect (Fig. 1e). They finally combine at two equidistant points from the center such that the middle sections form a loop (Fig. 1f). As the wave packet propagates further (passing through the focus), the reconnected structure breaks up again into two vortices but this time with the top and bottom loop segments switched (Fig. 1g,h).

**Figure 2:**
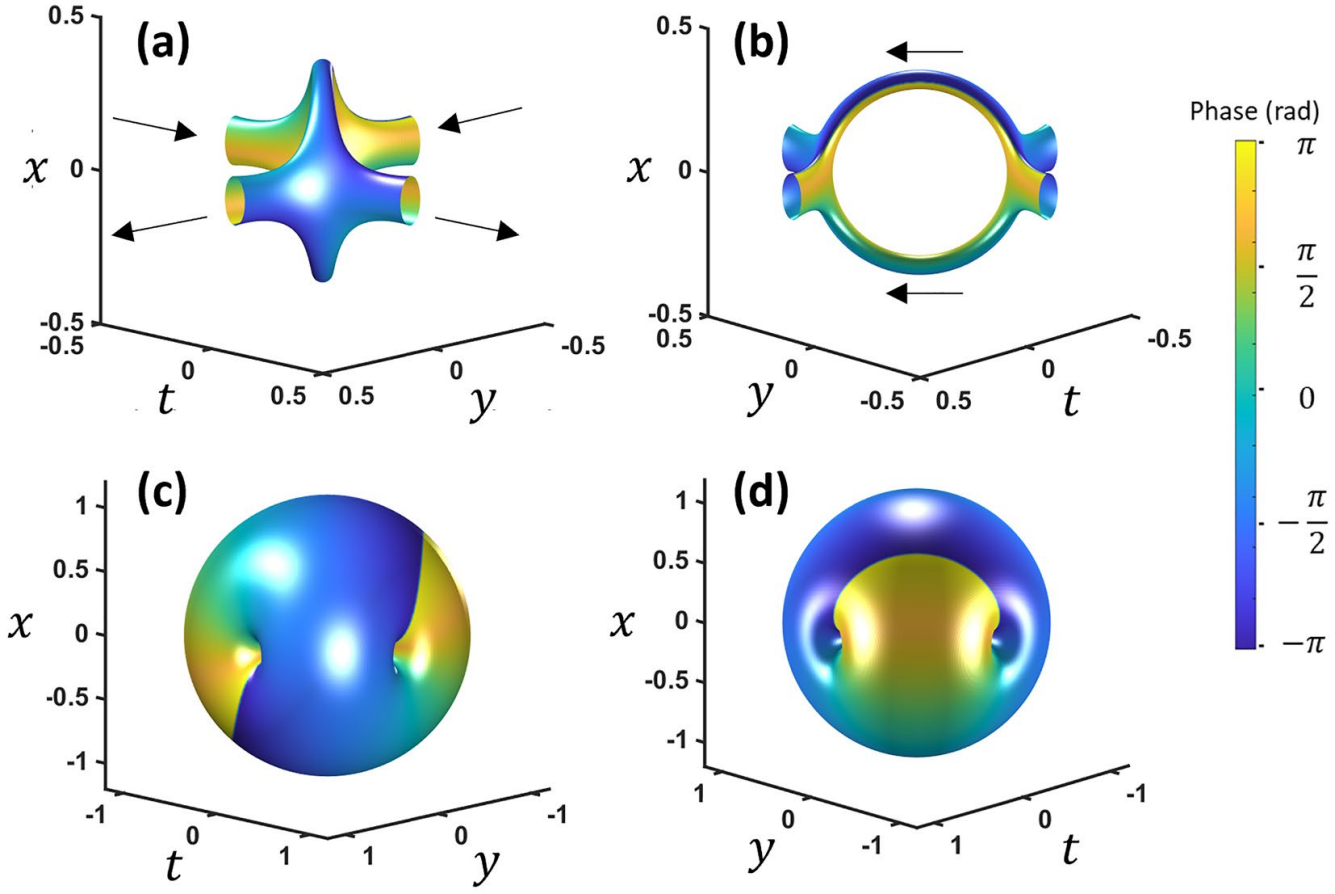
Different perspectives of the space–time domain after a 3D Fourier transform of two intersecting vortices. (**a**, **b**) show iso-intensity plots inside the wavepacket envelope while (**c**, **d**) show outside. The phase varies from − π (blue) to π (yellow). The arrows indicate the vortex direction. *x* and *y* units are in millimeters while *t* is in picoseconds.

The wave packet at the focus, which is the Fourier transform without quadratic phases, is $E(x,y,t) = \frac{1}{{w^{7} }}\left( {\frac{{w^{2} }}{2\pi } - x^{2} + 2lyt - i2\sqrt 2 x(ly + t)} \right)u$, where *u* is the Gaussian envelope of radius *w*. Rotating 45° in *y* − *t* to align with the loop such that *t*′ is perpendicular to the loop, looking near the loop plane (i.e.*t*^′^ near zero so *t*′^2^ <  < *y*^′2^ and *x*^2^), and using polar coordinates *r*^2^ = *x*^2^ + *y*^′2^, gives,

2 $$E(r,\phi ,t^{\prime } ) = \frac{1}{{w^{7} }}\left( {\frac{{w^{2} }}{2\pi } - r^{2} - i\sqrt 2 \cos (\phi )t^{\prime } } \right)u$$

which shows a spiral phase in *r* − t′ at a radius of $r = \frac{w}{{\sqrt {2\pi } }}$ Iso-intensity plots are shown in Fig. 2 for perspective inside and outside of the wave packet. The original two reconnected vortices appear to have arranged themselves with 180° rotational symmetry while the topological charge is one everywhere on the vortex path. In fact, this vortex reconnection structure resembles the theoretical work of Berry and Dennis^16^.

At the two connection points, there are no longer two distinct vortex lines. Instead, each vortex line is connected to two perpendicular vortex lines at the connection. For convenience, we will call this type of multi-vortex connection point a two-vortex connection (2-VC) in this manuscript to highlight each vortex is connected to two other perpendicular vortices.

**Figure 3:**
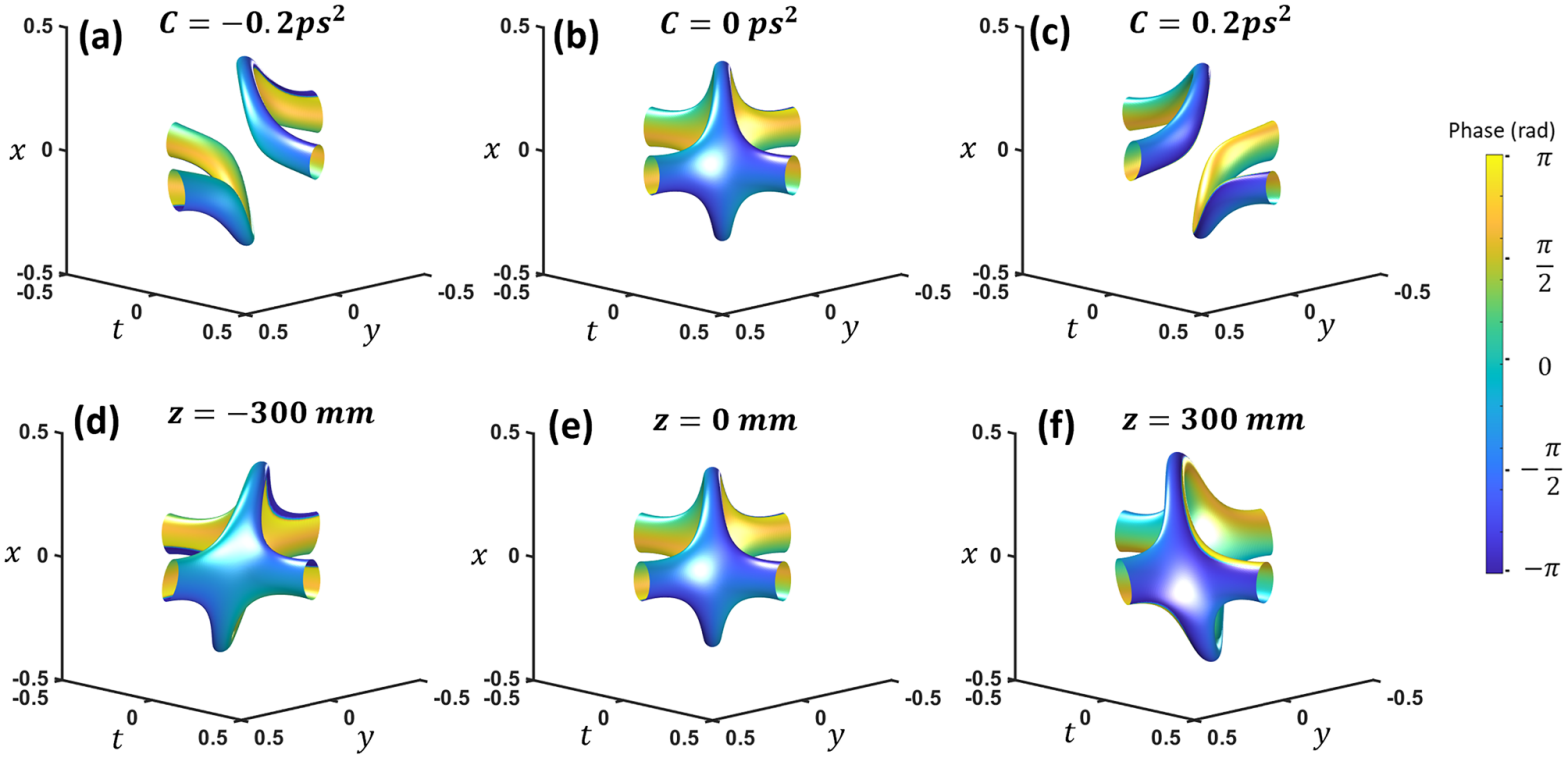
(**a**–**c**) Dispersion causes a sign dependent reconnection. (**d**–**f**) Propagation through a focus causes no reconnection for this orientation. *x* and *y* units are in millimeters while *t* is in picoseconds. See video 1 and 2 for chirp and focusing.

Diffraction and dispersion can be separately included in the model by breaking the original *A* term in Eq. (1) into separate components:

3 $$E = {\mathcal{F}}_{3D} \left\{ {\left( {k_{x} + \sqrt 2 i\omega } \right)\left( {k_{x} + \sqrt 2 iky} \right)\exp \left( { - \left( \frac{r}{w} \right)^{2} - i\pi \left( {k_{x}^{2} + k_{x}^{2} } \right)\lambda \left( {z - z_{0} } \right) - i\pi \omega^{2} C} \right)} \right\}$$

where *z*_0_ is the focal length of a lens while *C* governs the frequency quadratic phase (so-called chirp) to the pulse profile. With negligible spatial focusing, the effect of dispersion in Fig. 3a–c clearly shows the vortex reconnection. Two separate vortices merge into the loop structure with 2-VCs and separate into two different vortices. This reconnection would happen for a negatively chirped wave packet going through a positive dispersion media or vice versa. For the diffraction effect, we show the result of the wave packet through the focus of a lens without dispersion (Fig. 3d–f). This is equivalent to only the spatial Fresnel transform with the temporal Fourier transform. In this case, no reconnection clearly occurs. The 2-VCs remains intact while the loop rotates slightly along the common axis. However, reconnection from chirp and loop rotation from diffraction is only specific to this loop orientation. See the supplementary information for a more general description of loop orientation and reconnection.

**Figure 4:**
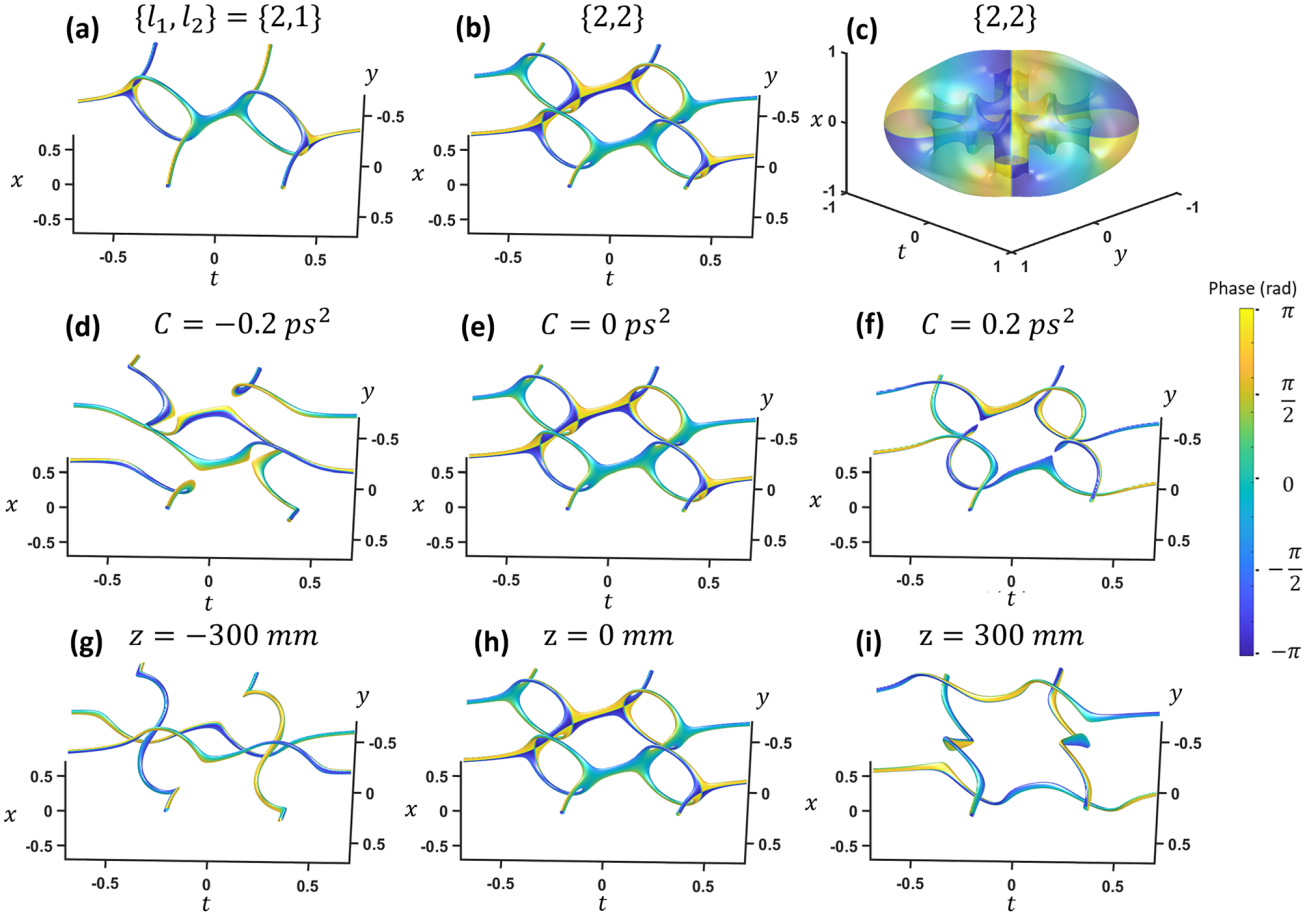
(**a**) Topological charge {2,1} and (**b**) {2,2} inside the envelope, and (**c**) {2,2} outside the wavepacket envelope. (**d**) Positive, (**e**) zero, and (**f**) negative dispersion on the same {2,2} state. Propagation of the {2,2} state (**g**) directly after a 300 mm focal length lens, (**h**) at the focus, and (**i**) two focal lengths after the lens. x and *y* units are in millimeters while *t* is in picoseconds. See video 3 and 4 for chirp and focusing.

Higher TC cases are shown in Fig. 4. At the focus, increasing the TC of one vortex creates an additional loop and two additional 2-VCs in the space–time domain (Fig. 4a). A typical iso-intensity profile plot is not adequate to see the vortex structures since the loops are farther from the center with low intensity. To show the internal vortex structure clearly, the iso-value of the intensity divided by some low pass filter intensity (|*E*_*filt*_|^2^ =|*E*|^2^/*lowpass*(|*E*|^2^) is plotted. Increasing the other vortex to TC = 2 results in in two additional loops (Fig. 4 (b)). The total number of loops is simply the multiplication of the two topological charges. This may be similar to flux-cutting arrays in superconductors^10,11^. The isointensity envelope surface, shown in Fig. 4c, reveals a TC equal to the sum (*i.e.* TC of *l*_1_ = 2 and *l*_2_ = 2 (which will be referred to {2,2}) results in a global TC = 4). Chirp causes a reconnection for each arm of each loop, breaking the array into four vortices of TC = 1(Fig. 4d–f). The top and bottom segments of each loop trade arms as chirp changes sign. The reconnections also happen with focusing (Fig. 4f,h), but with different reconnection pairs. These higher TC cases are similar to reference 14 which predicted instability of the higher-order topological loops by a perturbation. Perpendicular higher-order vortices evolve into multiple loops with TC = 1.

### Three-vortex

**Figure 5:**
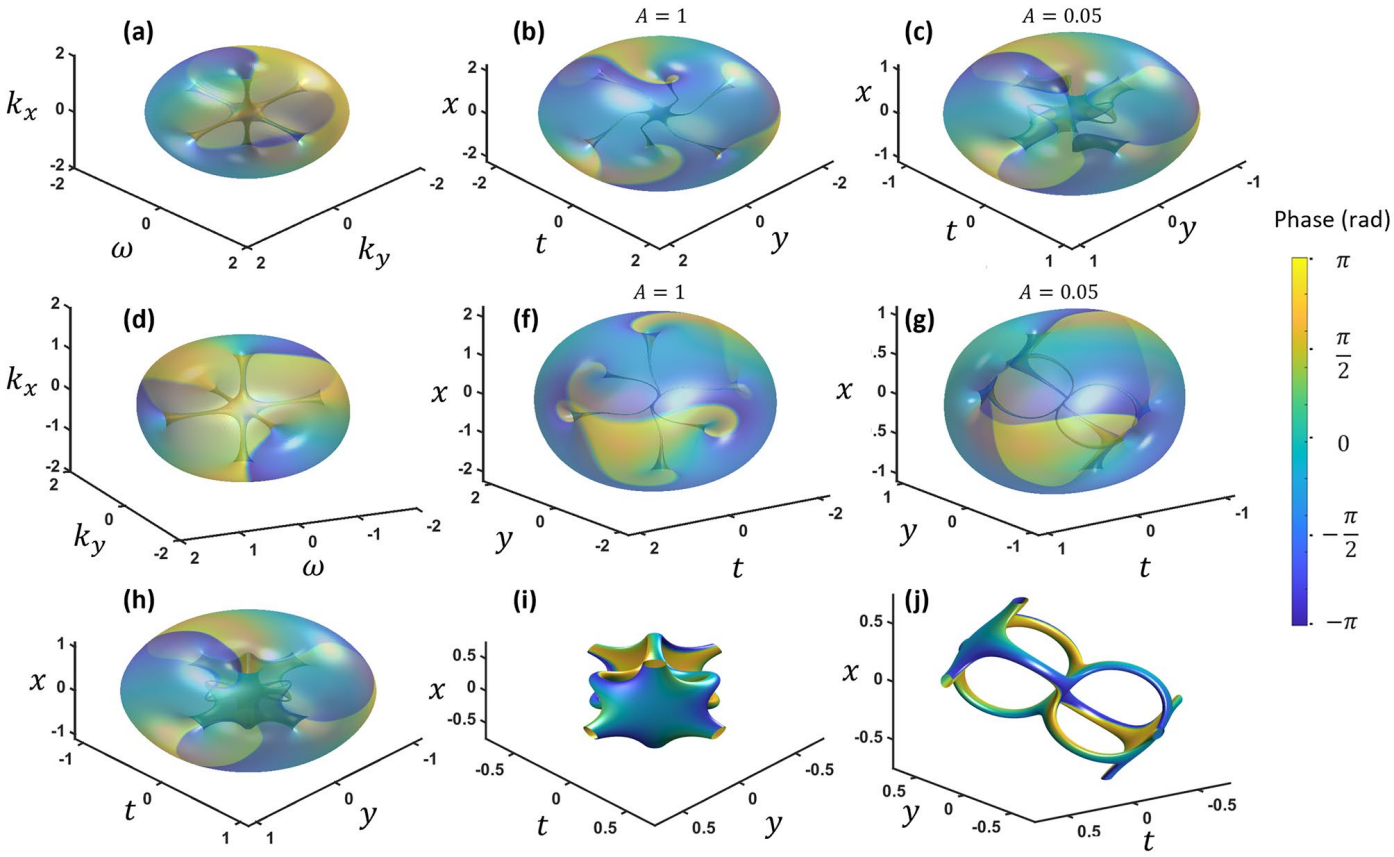
(**a**, **d**) Three perpendicular vortices in the frequency domain from different views. Space–time domain vortex behaviors (**b**, **f**) with a small propagation. (**c**, **g**) near focus. (**h**–**j**) at the focus. ${k}_{x}$ and ${k}_{y}$ units are in inverse millimeters and $\omega$ units are in THz, while *x* and *y* units are in millimeters and *t* is in picoseconds.

We can easily explore three-vortex reconnections by starting with three perpendicular vortices which are two perpendicular STOVs and one spatial vortex. The Fresnel transform will be governed by Eq. (4). Again, *A* will govern the converging/diverging of the wave packet in space and time simultaneously.

4 $$E = F_{3D} \left\{ {\left( {ik_{x} + \omega } \right)^{{l_{1} }} \left( {k_{x} + iky} \right)^{{l_{2} }} \left( {k_{y} + \omega } \right)^{{l_{3} }} \exp \left( { - \left( \frac{r}{w} \right)^{2} - i\pi Ar^{2} } \right)} \right\}$$

where *r*^2^ = *k*^2^_*x*_ + *k*^2^_*y*_ + ω^2^. We show the Fresnel transformation of a three-vortex case for *l*_1_ = 1, *l*_2_ = 1, and *l*_3_ = 1 in Fig. 5. Three vortices start to bend and eventually reconnect to form a unique loop pattern at the focus (Fig. 5h–j). At the focus, there are three connection points each consisting of three vortices. We will call this type of multi-vortex connection point a three-vortex connection (3-VC).

**Figure 6:**
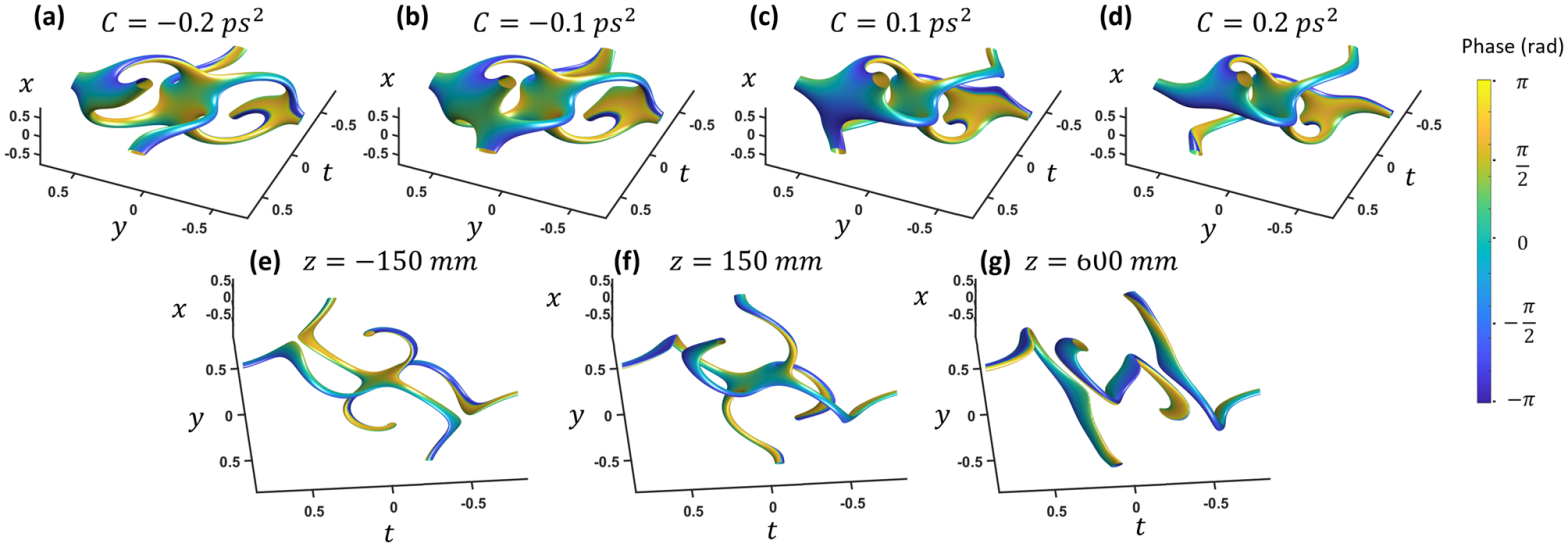
(**a**–**d**) The chirp of the wavepacket causes reconnection, each figure labeled with the corresponding amount of chirp. (**e**–**i**) Propagation due to a 300 mm lens, with z = 0 as the focus. *x* and *y* units are in millimeters while *t* is in picoseconds. See video 5 and 6 for chirp and focusing.

With diffraction or dispersion counted separately there are vortex reconnections and breaking up of some of the 3-VC’s (Fig. 6). With dispersion only, one vortex breaks off from the outer two points (Fig. 6a–d) as chirp is increased and the reconnections occur with the sign change of chirp. In this case, the two outer 3-VCs drop to 2-VCs while the inner 3-VC is stable until higher chirp is added (see video 5). The state shown in Fig. 6a,d is a combination of two of the 2-VC loops from the two-vortex case above, with an additional 3-VC connection at the center. With diffraction, the vortices break off one by one while departing from the focus, starting with the outer two 3-VC connections.

Considering the 2-VCs from the two-vortex case, having 3-VCs for three vortices is not a surprise.

**Figure 7:**
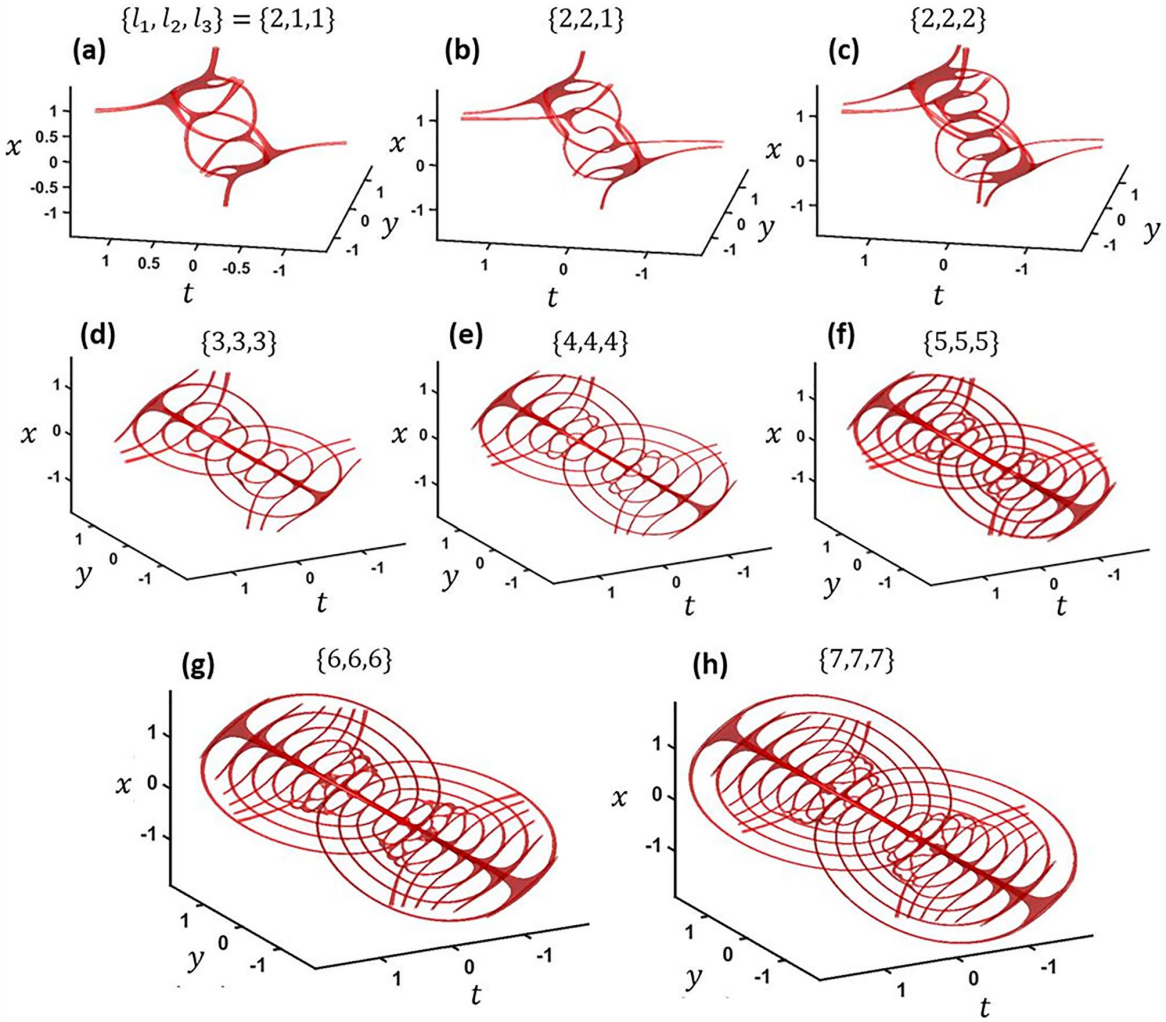
(**a**–**i**) The corresponding vortex structures at the focus for a three-vortex intersection of topological charge *l*_1_, *l*_2_ and *l*_3_. *x* and *y* units are in millimeters while *t* is in picoseconds.

However, the higher TC case becomes much more complicated and interesting for the three-vortex case. When increasing the TC of one vortex, an extra vortex appears and results in the middle 3-VC breaking up into two 2-VC points as shown in Fig. 7a. If the TC of a second vortex is increased to TC = 2 so that two are TC = 2 and one is TC = 1, i.e. {2,2,1}, a 3-VC appears in the center again (Fig. 7b). Bringing all of the three TCs to TC = 2 bumps the two 2-VCs up to 3-VCs so there are a total of six 3-VCs (Fig. 7c). Now each of the original spherical loops have another sphere nested inside and are connected to the adjacent opposite size sphere. Now, we will explore increasing the TC of all vortices together at the focus. With TC = {3,3,3}, each side now consists of three spheres (Fig. 7d). At TC = {4,4,4}, more spherical loops appear along with connections to loops in the adjacent set of spheres (Fig. 7e). As TC increases further, the trend continues with cascading nested loop structures (Fig. 7f–h). This cascading phenomenon may be similar to turbulence cascading between reconnected vortices in fluids^2^. For the turbulence cascade, small vortex threads appear between reconnected vortices, the threads reconnect, even smaller threads appear between those vortices, and this continues to smaller and smaller scales.

### Experiment

**Figure 8:**
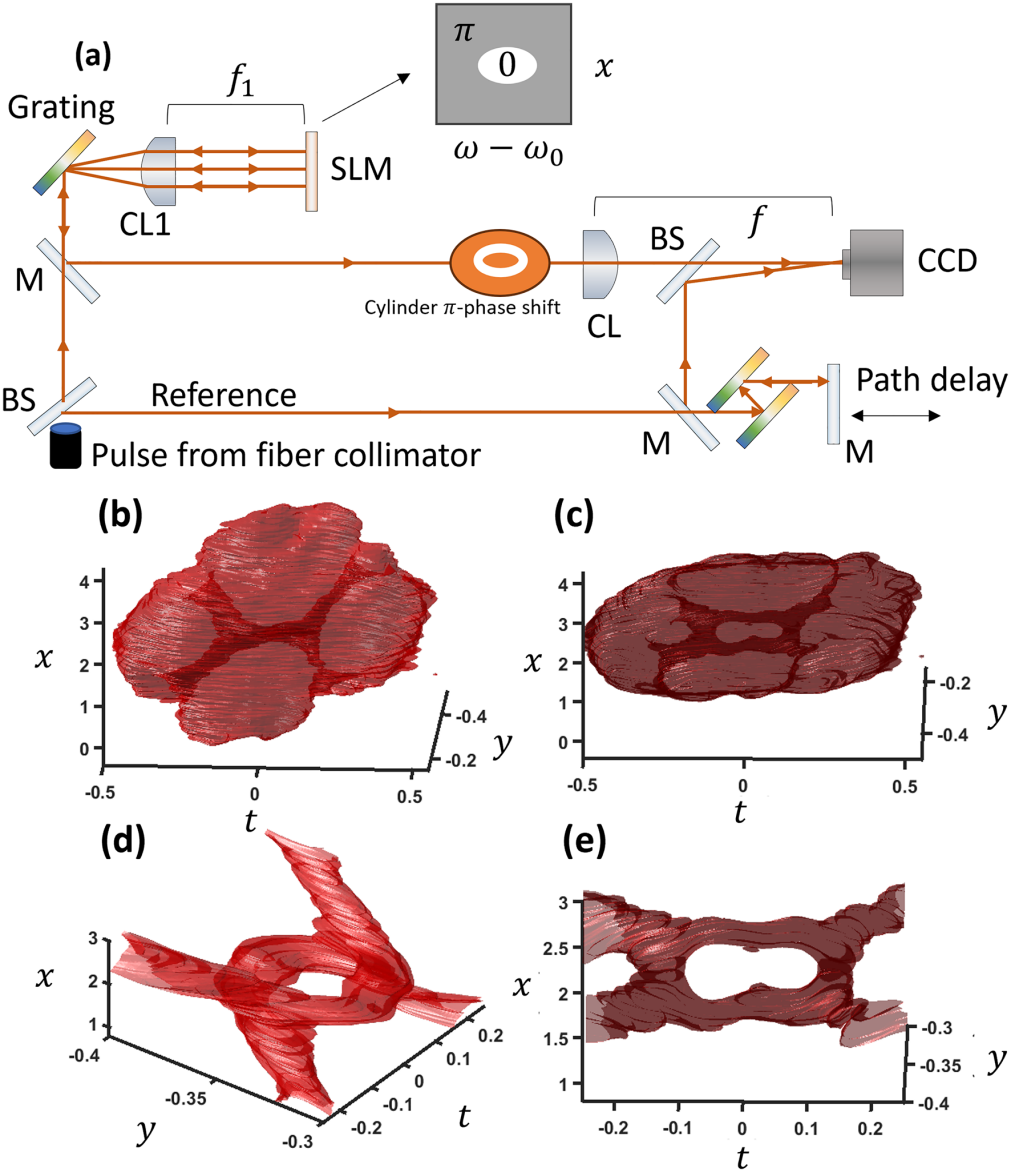
(**a**) Experimental setups for both path 1 and 2. (**b**–**c**) Experimental results at an iso-intensity value of 26% from different view angles. (**d**–**e**) The same results shown from inside the wavepacket envelope. *x* and *y* units are in millimeters while *t* is in picoseconds.

In the experiment, using two pulse shapers (*k*_*x*_ − ω, *k*_*y*_ − ω) or one pulse shaper and a beam shaper (*k*_*x*_ − ω, *k*_*x*_ − *k*_*y*_) would be an ideal setup for realizing two perpendicular vortices and therefore vortex reconnections. However, such an experimental setup could not be obtained in the present work. At the same time, the 3D feature of a tightly focused wave packet cannot be measured well since the CCD camera resolution is limited. Instead, a simpler but more restricted method was used which includes only a pulse shaper and a cylindrical lens using the fact that a specifically oriented 2D Fourier transform of the loop is just a cylindrical π-phase shift. The experimental setup and results are shown in Fig. 8. An SLM in a pulse shaper creates a cylindrical spatiotemporal phase shift, and a 45^∘^ rotated cylindrical lens turns this into a spatiotemporally oriented loop reconnection at the focus (see supplementary for further explanation). The cylindrical π-phase shift transitions to a two-vortex loop connection at the focus. The actual measurement process involves temporally scanning a reference pulse to find the intensity of the wavepacket at different delays^20^. Figure 8b gives the measured wave packet at the focus of the cylindrical lens, where x and y are in the cylindrical lens coordinates. The experimental result clearly shows a loop structure with 2-VCs for connected vortices.

## Conclusion

In this work, we show STOVs reconnect as vortices reconnect in many other fields. Vortices approach, reconnect, and depart from each other with specific evolution. In optical wave packets, very similar reconnection events develop when the wave packet is subjugated to dispersion or diffraction. We presented two perpendicular and three perpendicular vortices with various TC combinations that develop reconnection patterns in propagation. At the focus, two perpendicular vortices develop unique reconnection loop patterns. For the three vortices, especially higher TCs, cascading reconnections appear as cascaded loop structures which are similar to the turbulence cascading vortex reconnection in fluid.

We have shown experimental results for the TC = 1 two-vortex reconnection loop at the focus of a cylindrical lens. Future work should explore experimental setups using two or three phase modulation schemes such as two pulse shapers and one beam shaper. We believe that vortex reconnection in optical wave packets can be a useful tool for obtaining insight into vortex reconnections in other fields of physics.

## Methods

A mode-locked ytterbium ring cavity fiber laser was used to produce chirped pulses at ~ 1 ps in length centered at 1030 nm with a beamwidth of ~ 1.5 mm. The pulse is split into two paths, an object and reference pulse. The reference arm is sent through a dispersion compensating prism pair which brings the pulse duration down to ~ 60 fs and then to a delay arm for controlling the path length.

The pulse shaper is made of a 600/mm grating and a 100 mm cylindrical lens (CL1). The cylindrical π-phase shift parameters that resulted in the loop structure was elliptical with the small radius along the *x*-axis being 0.2 mm and the large radius along the ω axis is 0.6 mm. The cylindrical lens used for focusing (CL) has a focal length of 300 mm and the distance from the CL1 to CL is 1.1 m.

### Supplementary Information

Supplementary Information 1.

Supplementary Video 1.

Supplementary Video 2.

Supplementary Video 3.

Supplementary Video 4.

Supplementary Video 5.

Supplementary Video 6.

## Data Availability

Data underlying the results presented in this paper are available from the corresponding authors upon reasonable request.
