# Supplementary material for: Spatiotemporal optical vortex reconnections of multi-vortices
Source: Sci Rep. 2024 Mar 6;14:5483. doi: 10.1038/s41598-024-54216-4 (PMC10914776; doi:10.1038/s41598-024-54216-4)
Supplement: Supplementary file 1 — Supplementary Information 1. [file 41598_2024_54216_MOESM1_ESM.docx]

Supplementary information:

Spatiotemporal optical vortex reconnections of multi-vortices

1. Orientation and chirp/diffraction

Most orientations of chirp or diffraction with respect to the loop cause reconnection However, there are specific orientations that result in loop rotation instead. To define the loop orientation, Figure S1 illustrates the coordinate attached the loop. $\gamma$ is the axis along the two connection points while $\beta$ is the axis perpendicular to the circle of the loop and $\alpha$ is the axis perpendicular to$\beta$ and $\gamma$ . In terms of the theoretical example shown in the manuscript, the $\alpha-\beta$ coordinates are the laboratory $y-t$ axes rotated by 45 degrees (bottom blue axes) while the $x$ axis is the $\alpha$ axis (top blue axis). For that case, this means one of blue arrows in in the $\beta-\gamma$ plane would be the propagation direction.

It turns out that the 1D quadratic phase along $\gamma$ (in the red arrow direction) results in a loop rotation without a reconnection. If there are two quadratic phases, one in the $\alpha$ direction (top blue arrow) and another in any of the two bottom blue arrow directions, the loop rotation will occur instead of reconnection. This is equivalent to focusing only case in the main manuscript. Since the spatially focusing case induces quadratic phases on a vertical blue axis and one of the horizontal blue axes, the loop rotation without reconnection will occur. So far, all other cases have ended up with reconnections based on our simulations. The single-axis quadratic phase can be chirp or 1D focusing.


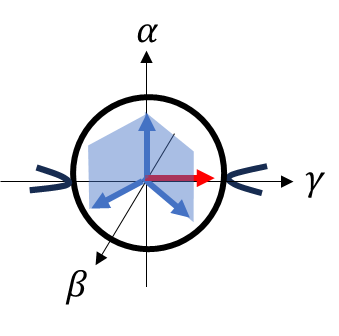


**Figure S1:** Loop structure with relevant coordinate systems to define the loop orientation.

If a 1D quadratic phase is applied in the $\beta$-axis direction, a reconnection occurs. With a 1D Fourier transform of $\beta$ spatial coordinate, the 3D structure eventually collapses to a 2D cylinder structure.


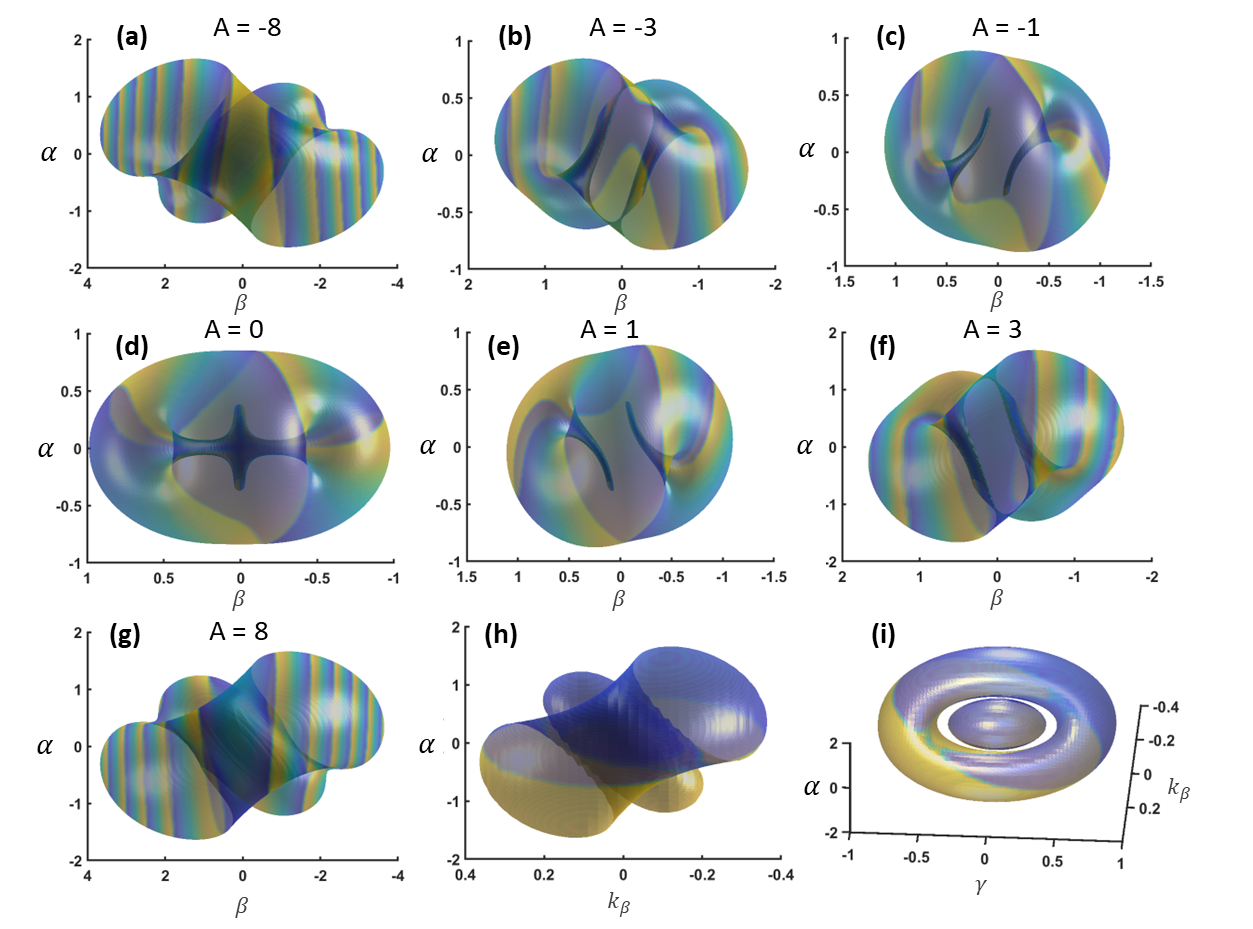


Figure S2: The example of reconnection with the quadratic phase along the $\beta$-axis. The Fresnel transforms with increasing A are shown in (a)-(g). h) and i) show the 1D Fourier transform of (g).

1. Experimental relationship to model

Figure S2 shows that a $\beta$-axis Fourier Transform collapses the 3D loop into a 2D cylindrical structure (Figure S2(g)). Such cylindrical 2D structures can be generated experimentally with only one phase applied in a pulse shaper. Therefore, by the inverse 1-D Fresnel transform (which is the 1D focusing in $\beta$ -direction of Figure S2(g)), we can obtain the reconnection loop structure in Figure S2(d).

Now, the spatiotemporally oriented loop in figure S2(d) is:

$$E\propto\left( \alpha^{2}+\gamma^{2}-\beta^{2}-r_{o}^{2}+i2\alpha\beta\right)u [S1]$$

and take the Fourier transform along the $\beta$ -axis, we arrive to an equation of the form

$$\mathcal{F}_{\beta}^{-1}\left\{ E \right\}\propto\left( \alpha^{2}+\gamma^{2}+ak_{\beta}^{2}-r_{1}^{2}+b2\alpha k_{\beta} \right)u^{'}. [S2]$$

If $a$=$b$=1, then the dimensions can be reduced by rotating axes $\alpha-k_{\beta}$ by 45 degrees. To tie this to our experiment, we label these new axes $x-k_{y}$to represent laboratory coordinates. Additionally, we can replace $\gamma$ axis with the time axis,$t,$

$$R_{\alpha k_{\beta}}\left\{ \mathcal{F}_{\beta}^{-1}\left\{ E \right\} \right\}\propto\left( x^{2}+t^{2}-r_{1}^{2} \right)u^{'}. [S3]$$

From Equation S3 it is clear that the amplitude has a sign change when $x^{2}+t^{2}=r_{1}^{2}$. In other words, there is a $\pi$phase shift across the intensity null. This phase is plotted in Figure S3 (b) next to the intensity in Figure S3 (a).


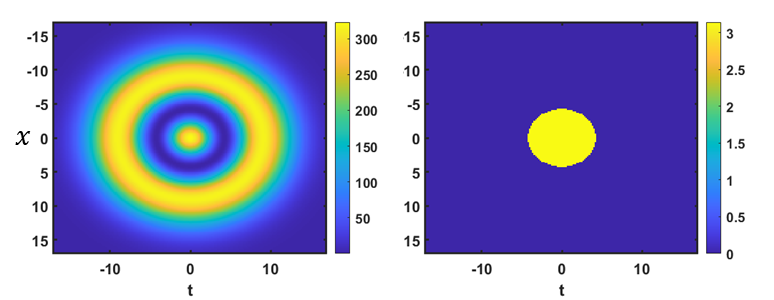


**Figure S3:** **(a)** Intensity and $\left( \boldsymbol{b} \right)$ phase of the two-vortex loop connection after a $\beta$-axis Fourier transform and $\alpha-k_{\beta}$ rotation.

Therefore, if we apply a cylindrical $\pi$ phase shift on the pulse shaper then after exiting, with sufficient propagation, the wavepacket will have a cylindrical intensity null with the cylindrical $\pi$ phase shift (Equation S3) in $x-t$ coordinates. To turn this into the loop connection we use a cylindrical lens. The cylinder will be oriented spatiotemporally in $x-t$ so this means we have to orient the cylindrical lens at a 45 degree angle in the $x-y$plane (where here $y$ is effectively $k_{y}$). Rotating to the lens coordinates allows us to turn Equation S3 back to S2 and the focusing takes S2 back to Equation S1 which is the reconnection loop structure.

1. Cross sections

To help further understand what is occurring during the reconnection, we show cross section of intensity and phase in Figure S4 and S5. Here, we use the example from the manuscript of the STOV and spatial two-vortex reconnection occurring with propagation through dispersive media. Cross sections of at the plane $\gamma=0$ and $\gamma=0.32$ plane are shown in Figure S4 for $-0.2 ps^{2}$, $0 ps^{2}$, and $0.2 ps^{2}.$ Figure S4 (a)-(f) shows the $\gamma=0$ plane where the top and bottom part of the loop occur at zero chirp. The points in this plane do not move during the reconnect process, although is it no longer the top or bottom of the loop that is piercing the plane. At $\gamma=0.32$ is one of the planes where the two vortices connect (Figure S4(d)-(l). Here the intensity is a single point and the phase a single spiral at zero chirp. But the point and spiral phase breaks into two with positive or negative chirp as the two vortices move away.


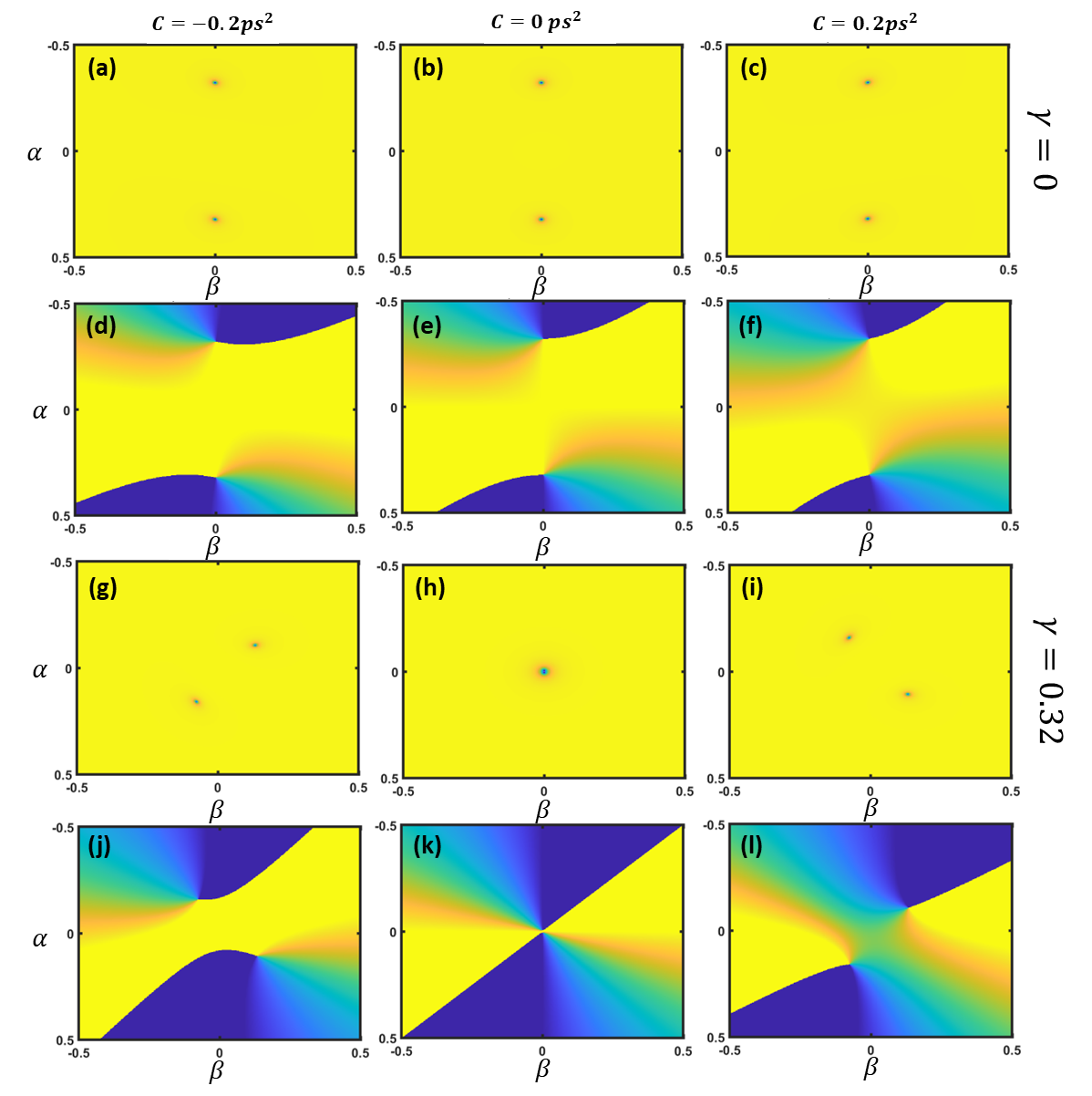


**Figure S4:** $\gamma=0$ cross section **for (a)-(c)** intensity and **(d)-(f)** phase of the STOV and spatial two-vortex reconnection process with a chirped wavepacket traveling through dispersive media. $\gamma=0.32$ cross section showing one of the connection points for **(g)-(i)** intensity and **(j)-(l)** phase for the same process. Phase is from $-\pi/2$ (blue) to $\pi/2$(yellow).

The $\beta=0$ plane is shown in Figure S5 for intensity (a)-(c) and phase (d)-(f). The ring is clearly shown at zero chirp, but it breaks up at with positive or negative chirp. The $\alpha=0$ plane is shown in Figure S5 (g)-(l). This shows the arms of the vortices connect at zero chirp, but break apart with positive or negative chirp.


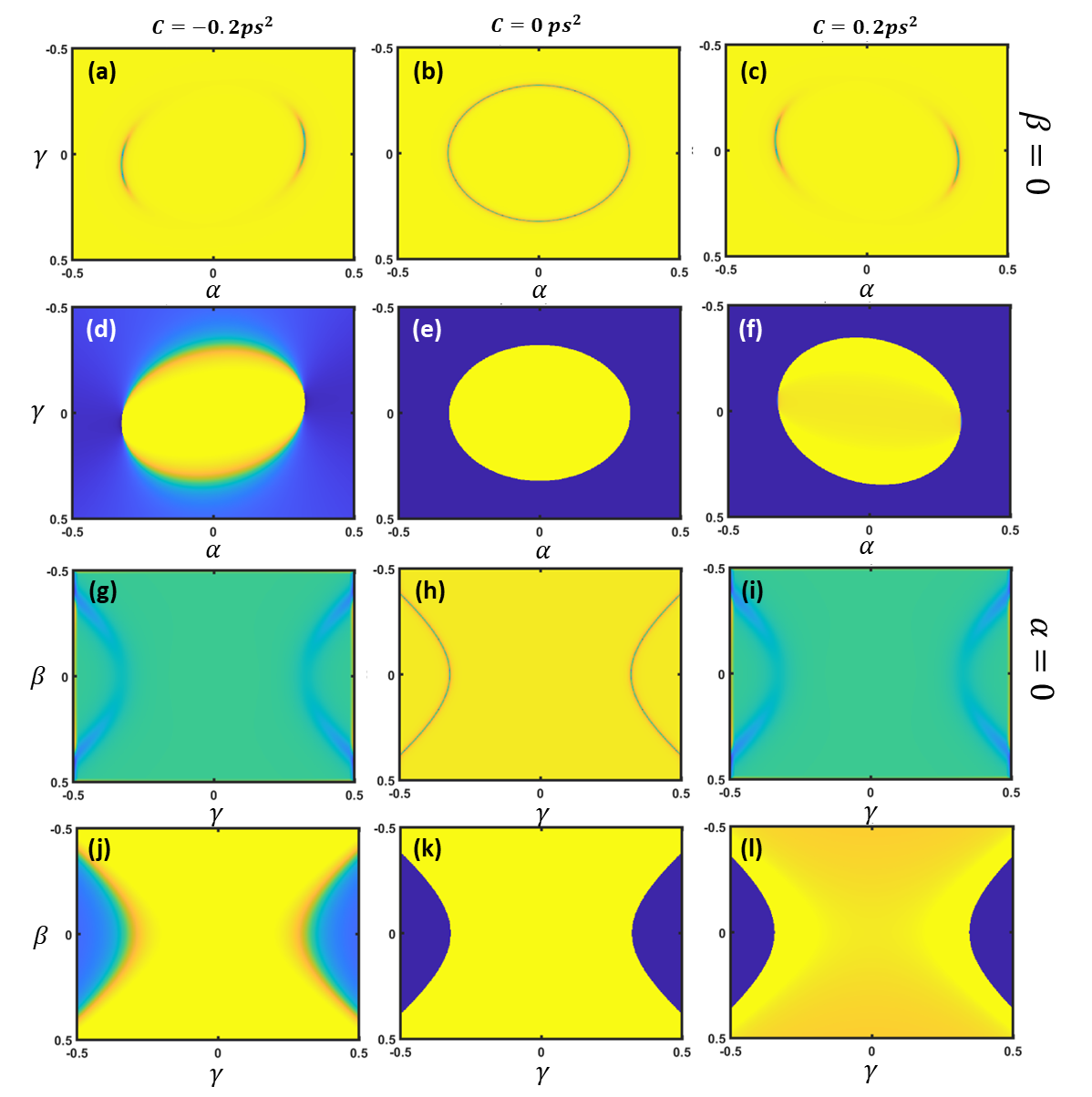


**Figure S5:** $\beta=0$ cross section **for (a)-(c)** intensity and **(d)-(f)** phase of the STOV and spatial two-vortex reconnection process with a chirped wavepacket traveling through dispersive media. $\alpha=0$ cross section for **(g)-(i)** intensity and **(j)-(l)** phase for the same process. Phase is from $-\pi/2$ (blue) to $\pi/2$(yellow).

Supplementary videos

Video 1. The vortex reconnection when the chirped wavepacket travels through a dispersive media.

Video 2. The vortex reconnection when the wavepacket travels through the 300 mm focal length spherical lens.

Video3. The vortex reconnection when the chirped wavepacket travels through a dispersive media. Both vortices have topological charge of two.

Video 4. The vortex reconnection when the wavepacket travels through the 300 mm focal length spherical lens. Both vortices have topological charge of two.

Video 5. A three-vortex reconnection when the chirped wavepacket travel through a dispersive media.

Video 6. A three-vortex reconnection when the wavepacket travels through the 300 mm focal length spherical lens.
