# Supplementary figures and images for: Spatiotemporal optical vortex reconnections of multi-vortices
Source: Sci Rep. 2024 Mar 6;14:5483. doi: 10.1038/s41598-024-54216-4 (PMC10914776; doi:10.1038/s41598-024-54216-4)

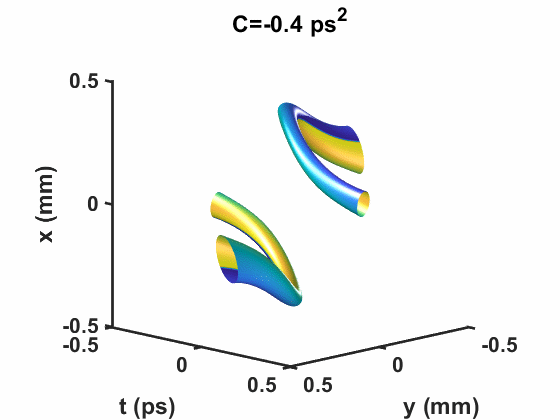

Supplement: Supplementary file 2 — Supplementary Video 1. [file 41598_2024_54216_MOESM2_ESM.gif]

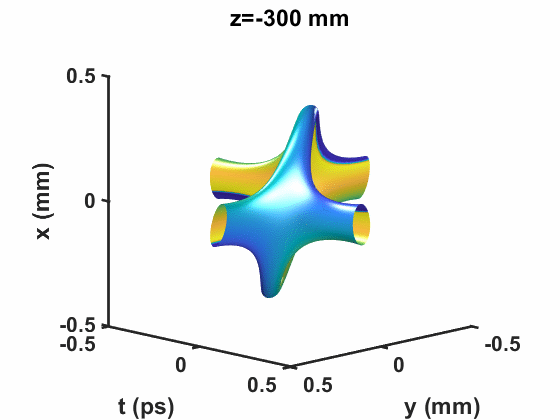

Supplement: Supplementary file 3 — Supplementary Video 2. [file 41598_2024_54216_MOESM3_ESM.gif]

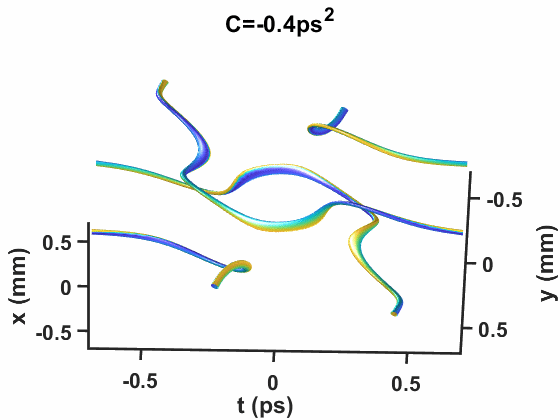

Supplement: Supplementary file 4 — Supplementary Video 3. [file 41598_2024_54216_MOESM4_ESM.gif]

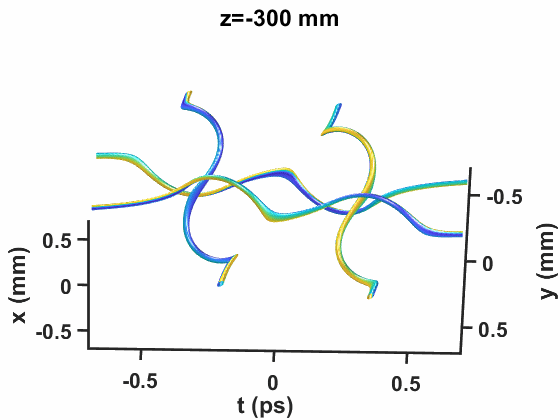

Supplement: Supplementary file 5 — Supplementary Video 4. [file 41598_2024_54216_MOESM5_ESM.gif]

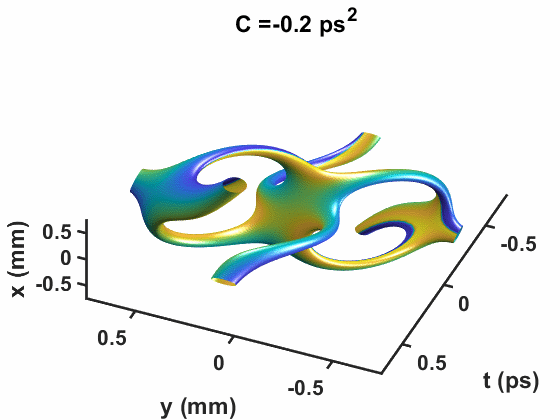

Supplement: Supplementary file 6 — Supplementary Video 5. [file 41598_2024_54216_MOESM6_ESM.gif]

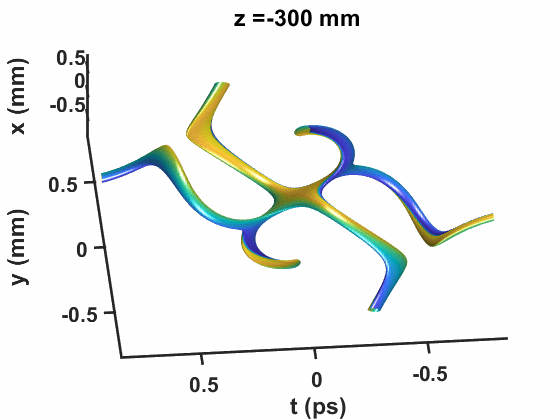

Supplement: Supplementary file 7 — Supplementary Video 6. [file 41598_2024_54216_MOESM7_ESM.gif]
